# Maintained therapeutic effect of revefenacin over 52 weeks in moderate to very severe Chronic Obstructive Pulmonary Disease (COPD)

**DOI:** 10.1186/s12931-019-1187-7

**Published:** 2019-10-30

**Authors:** James F. Donohue, Edward Kerwin, Sanjay Sethi, Brett Haumann, Srikanth Pendyala, Lorna Dean, Chris N. Barnes, Edmund J. Moran, Glenn Crater

**Affiliations:** 1 Pulmonary Medicine, UNC School of Medicine, Chapel Hill, NC USA; 2 Clinical Research Institute of Southern Oregon, PC, Medford, OR USA; 3 University at Buffalo, State University of New York, Buffalo, NY USA; 4 Theravance Biopharma US, Inc., 901 Gateway Boulevard, South San Francisco, CA 94080 USA

## Abstract

**Background:**

Revefenacin is a long-acting muscarinic antagonist that was recently approved for the nebulized treatment of chronic obstructive pulmonary disease (COPD). Although shorter duration studies have documented the efficacy of revefenacin in COPD, longer-term efficacy has not been described. In a recent 52-week safety trial, revefenacin was well tolerated and had a favorable benefit-risk profile. Here we report exploratory efficacy and health outcomes in patients receiving revefenacin 175 μg or 88 μg daily during the 52-week trial.

**Methods:**

In this randomized, parallel-group, 52-week trial (NCT02518139), 1055 participants with moderate to very severe COPD received revefenacin 175 μg or 88 μg in a double-blind manner, or open-label active control tiotropium.

**Results:**

Over the 52-week treatment period, both doses of revefenacin, as well as tiotropium, elicited significant (all *p* < 0.0003) improvements from baseline in trough forced expiratory volume in 1 s (FEV_1_). The trough FEV_1_ profile (least squares mean change from baseline) for revefenacin 175 μg ranged from 52.3–124.3 mL and the trough FEV_1_ profile for tiotropium ranged from 79.7–112.8 mL. In subgroup comparisons, the effect of revefenacin on trough FEV_1_ was comparable in patients taking concomitant long-acting β-agonists, with or without inhaled corticosteroids, with patients who were not taking these medications. There were statistically significant (*p* < 0.05) improvements in all measured health status outcomes (evaluated using St. George’s Respiratory Questionnaire, COPD Assessment Test, Clinical COPD Questionnaire and Baseline and Transition Dyspnea Index) from 3 months onward, in all treatment arms.

**Conclusions:**

Significant sustained improvements from baseline in trough FEV_1_ and respiratory health outcomes were demonstrated for 175-μg revefenacin over 52 weeks, further supporting its use as a once-daily bronchodilator for the nebulized treatment of patients with COPD.

**Trial registration:**

NCT02518139; Registered 5 August 2015.

## Background

Treatment with bronchodilators is central to the management of chronic obstructive pulmonary disease (COPD). Treatment guidelines produced by the Global Initiative for Chronic Obstructive Lung Disease (GOLD) recommend inhalation therapy with long-acting muscarinic antagonist (LAMA) or long-acting β-agonist (LABA) bronchodilators as first-line therapy to address COPD symptoms and prevent exacerbations [1]. The use of an inhaled corticosteroid (ICS) in combination with LABA or LABA/LAMA therapy is recommended for patients experiencing exacerbations despite the use of bronchodilator therapy [1].

Until recently, LAMAs were not available in a nebulized form. The LAMA glycopyrrolate bromide was recently approved (2017) for twice-daily nebulized delivery via a custom-designed electronic vibrating mesh nebulizer (eFlow®; Lonhala® Magnair®, Sunovion; Marlborough, MA, USA) [2]. Revefenacin, a novel, lung-selective LAMA, was approved by the US Food and Drug Administration (FDA) in November 2018 at a 175 μg dose, for once-daily nebulized delivery via a standard jet nebulizer for the maintenance treatment of COPD [3]. Beyond the differences in frequency and method of administration, revefenacin is a different molecular class than glycopyrrolate bromide—it is a tertiary amine, not a quaternary ammonium compound. Thus, it is a different molecular class from all the inhaled muscarinic antagonists (glycopyrrolate bromide, tiotropium bromide [tiotropium], umeclidinium bromide, aclidinium and ipratropium bromide) available to date. Revefenacin was designed to produce sustained local bronchodilation with minimal systemic drug exposure [4, 5], and as a result appears to have lower potential for systemic anti-muscarinic side effects than quaternary ammonium compounds [6].

Replicate 12-week pivotal phase 3 trials demonstrated significant bronchodilation in patients with moderate to very severe COPD taking once-daily inhaled revefenacin at doses of 175 μg and 88 μg, with or without concomitant LABA/ICS therapy [7]. The safety and tolerability of revefenacin over 52 weeks has been presented previously [8]. Here we report the maintained therapeutic effect of revefenacin and improvement in health outcomes versus tiotropium over the course of 52 weeks in patients with moderate to very severe COPD.

## Methods

### Study design and conduct

This was a phase 3, randomized, partially double-blinded, parallel-group 52-week trial (NCT02518139). The trial was conducted in accordance with the principles of the International Council on Harmonisation of Technical Requirements for Pharmaceuticals for Human Use guideline for good clinical practice [9], and the code of ethics of the World Medical Association’s Declaration of Helsinki [10], and all patients provided written informed consent.

### Patients and treatments

Study design, participants and treatments have been described previously [8]. Briefly, patients were required to meet the criteria for moderate-to-severe COPD, which included < 0.7 postipratropium forced expiratory volume in 1 s (FEV_1_)/forced vital capacity ratio and < 80% postipratropium FEV_1_ of predicted normal but at least 700 mL [11]. Patients were excluded if they had significant respiratory disease other than COPD, elevated cardiovascular risk (e.g.*,* myocardial infarction or unstable angina within the previous 6 months, unstable or life-threatening arrhythmia requiring intervention in the previous 3 months, or New York Heart Association Class IV heart failure) or exhibited a clinically significant abnormality in 12-lead electrocardiogram at screening, or uncontrolled hypertension, hypercholesterolemia, or type 2 diabetes. In addition, patients were ineligible for participation if they had been hospitalized for COPD or pneumonia within 8 weeks of screening or had used systemic corticosteroids or antibiotics within 6 weeks of screening. Patients were equally randomized to receive revefenacin 175 μg or 88 μg, or tiotropium 18 μg, for 52 weeks. Revefenacin dose was assigned in a double-blind manner, and the drug was administered using a standard jet nebulizer (PARI LC® Sprint; Midlothian, VA, USA). Tiotropium was administered open label using the HandiHaler® device (Spiriva® HandiHaler; Boehringer Ingleheim, Ridgefield, CT, USA). Stratified randomization was used to randomize patients according to their reversibility status to ipratropium and use of concomitant LABA or LABA/ICS treatment at screening.

### Assessments and endpoints

The primary endpoint results (safety and tolerability of revefenacin as a treatment for COPD over 52 weeks) were previously reported [8]. Here we report exploratory efficacy and health outcomes endpoints and use of rescue medication, all of which were evaluated as change from baseline (and not as between-treatment comparison). These exploratory endpoints included the change in trough FEV_1_, changes in health outcomes using general and COPD-specific respiratory symptom rating instruments (St. George’s Respiratory Questionnaire [SGRQ], COPD Assessment Test [CAT], Clinical COPD Questionnaire [CCQ], EXAcerbation of Chronic Pulmonary Disease Tool Patient Related Outcome [EXACT-PRO], Baseline and Transition Dyspnea Index [BDI/TDI]), and concomitant use of rescue medications, all over 12 months.

Change from baseline in FEV_1_ was measured prior to drug administration at 1, 3, 6, 9 and 12 months (Days 29, 92, 183, 274 and 365). Patient-reported outcomes (SGRQ, CAT, CCQ, BDI/TDI) were assessed in the clinic on Days 1, 92, 183, 274 and 365; EXACT-PRO [12], a patient-reported outcome electronic diary designed to count and characterize symptom-defined exacerbations was used by patients each night. Treatment satisfaction was measured using a non-validated treatment satisfaction questionnaire at screening and on Day 365.

The minimal clinically important difference (MCID) for each assessed outcome was used to determine response to treatment (responders). For SGRQ, a reduction in mean baseline score of 4 points is considered clinically relevant. Although there is no generally accepted MCID for the CAT, a change of 2 points appears to be clinically relevant and correlates well with the MCID of other validated health status measures. For CCQ, the validated MCID is 0.4, and for TDI the benchmark is a difference between groups in total score of ≥1 point. For this study, patients who met or exceeded the MCID on each measure were considered responders.

Patients documented whether or not they used the albuterol metered dose inhaler as rescue medication over each 24-h period in a study diary. In addition, the number of puffs from the inhaler was recorded by study site personnel at each clinic visit. Rescue albuterol use (number of puffs per day) was averaged over the 12-month treatment period and reported for Months 1, 3, 6, 9 and 12. Data for analysis were drawn from inhaler counter totals recorded in the electronic case report form and not the self-reported data, as inhaler counters were considered to be more reliable.

### Statistical analyses

The exploratory analyses were performed using the intent-to-treat (ITT) population, defined as all randomized patients who received at least one dose of study drug and had at least one postbaseline FEV_1_ measurement.

Trough FEV_1_ was defined as the mean of the − 45- and − 15-min pre-dose spirometry assessments and evaluated using a repeated measures mixed-effect model (RMMM) model. Treatment group, smoking status, ipratropium reversibility status, concomitant LABA/ICS use at baseline, sex and age at baseline (≤65 years or > 65 years) were fixed effects. A covariate for baseline FEV_1_ was included. A time effect and its interaction with treatment and baseline FEV_1_, as well as a random effect for subject nested within site/center was included in the model. Within-subject correlation was modelled using an unstructured covariance. Pre-specified subgroup analyses were performed for the change from baseline in trough FEV_1_ among patients who used a LABA-containing product or ICS, were ≥ 65 years old and were current smokers.

SGRQ, CAT and BDI/TDI were summarized as continuous measures using absolute values and change from baseline at each visit, and as a proportion using a responder definition. The CCQ data were summarized as continuous measures using absolute values and change from baseline at each visit.

Treatment satisfaction was summarized using descriptive statistics. The number of puffs of rescue medication and number of rescue medication–free days were analyzed using a RMMM model with the same independent variables as those described for FEV_1_.

The primary endpoint of this study was to assess long term safety and tolerability of revefancin, and therefore was not designed or powered to show differences between treatments.

## Results

### Patients

Of the 1060 patients randomized to treatment, 1055 received at least one dose of study drug. The ITT analysis set comprised 1020 patients (revefenacin 175 μg, *n* = 319; revefenacin 88 μg, *n* = 350; tiotropium, *n* = 351). Patient demographics and baseline characteristics in the ITT population were similar between treatment groups (Table 1).

**Table 1:** Baseline patient demographics and clinical characteristics (ITT population)

Characteristic	Revefenacin 88 μg (*n* = 350)	Revefenacin 175 μg (*n* = 319)	Tiotropium 18 μg (*n* = 351)
Age, y, mean (SD)	64.2 (9.37)	64.5 (8.61)	64.9 (8.91)
Sex, male, *n* (%)	197 (56.3)	188 (58.9)	211 (60.1)
Race, white, *n* (%)	324 (92.6)	294 (92.2)	326 (92.9)
BMI, kg/m^2^, mean (SD)	28.9 (6.6)	29.0 (6.6)	28.8 (6.3)
Current smoker, *n* (%)	163 (46.6)	140 (43.9)	164 (46.7)
Concurrent COPD medication use, *n* (%)			
ICS	191 (54.6)	165 (51.7)	187 (53.3)
LABA or ICS/LABA	175 (50.0)	158 (49.5)	177 (50.4)
ICS/LABA	170 (48.6)	146 (45.8)	172 (49.0)
COPD duration, y, mean (SD)	9.3 (6.98)	8.7 (5.92)	9.5 (6.84)
2011 GOLD^a^ category, *n* (%)			
A	31 (8.9)	20 (6.3)	23 (6.6)
B	184 (52.6)	165 (51.7)	183 (52.1)
C	7 (2.0)	7 (2.2)	5 (1.4)
D	128 (36.6)	123 (38.6)	136 (38.7)
Unknown	0	4 (1.3)	4 (1.1)
2011 GOLD^a^ airflow limitation category, *n* (%)			
2	222 (61.0)	201 (60.0)	210 (59.0)
3	122 (33.5)	109 (32.5)	124 (34.8)
4	20 (5.5)	25 (7.5)	22 (6.2)
Postipratropium percent predicted FEV_1_, %, mean (SD)	54.29 (14.1)	53.75 (14.8)	53.15 (14.2)
Postipratropium FEV_1_ to FVC ratio, mean (SD)	0.54 (0.10)	0.53 (0.10)	0.53 (0.10)
Baseline FEV_1_, L, mean (SD)	1.34 (0.5)	1.34 (0.5)	1.32 (0.5)
Proportion of patients with baseline mMRC ≥ 2, *n* (%)	181 (51.7)	170 (53.3)	180 (51.3)
Proportion of patients with baseline CAT ≥ 10, *n* (%)	312 (89.1)	288 (90.3)	319 (90.9)
Number of COPD exacerbations in prior year, *n* (%)			
0	264 (75.4)	242 (75.9)	271 (77.2)
1	59 (16.9)	50 (15.7)	52 (14.8)
≥ 2	27 (7.7)	27 (8.5)	28 (8.0)

^a^2011 GOLD criteria. (Global Initiative for Obstructive Lung Disease. Global strategy for the diagnosis, management and prevention of chronic obstructive pulmonary disease. 2011. http://goldcopd.com)

*BMI* body mass index, *CAT* COPD assessment test, *COPD* chronic obstructive pulmonary disease, *FEV*_*1*_ forced expiratory volume in 1 s, *FVC* forced vital capacity, *GOLD* Global Initiative for Chronic Obstructive Lung Disease, *ICS* inhaled corticosteroids, *ITT* intent-to-treat, *LABA* long-acting beta agonist, *mMRC* modified Medical Research Council, *SD* standard deviation

Over the year-long study, there were more withdrawals from the revefenacin treatment arms (39–43%) than from the tiotropium arm (26%), which led to wider confidence intervals (CIs) and more variable data at the 9- and 12-month time points for all treatment arms (Table 2). A higher percentage of subjects in the revefenacin groups discontinued study drug in the first 3 months (60/335 [17.9%] and 50/368 [13.6%] in the revefenacin 175 μg and 88 μg groups, respectively) compared with the tiotropium group (25/357, [7.0%]). There were 2–3 times more withdrawals from the revefenacin arms in the “withdrawal by subject” category compared with the tiotropium arm (Table 2). A review of those subjects reported as discontinuing the treatment period for the reason “withdrawal by subject” indicated that patients did not appear to drop out of the revefenacin arms of the study due to lack of efficacy. A post hoc analysis of withdrawal by FEV_1_ response showed, that patients in the revefenacin 175 μg group had greater FEV_1_ improvements from baseline versus the tiotropium group at various time points (Additional file 1: Table S1).

**Table 2:** Patients withdrawals during the 1-year treatment period

	AE, *n* (%)	Lost to follow-up, *n* (%)	Physician decision, *n* (%)	Protocol deviation, *n* (%)	Withdrawal by subject, *n* (%)	Other, *n* (%)	Total, *n* (%)
Revefenacin 88 μg	(*n* = 368)						
Days 1–30	11 (3.0)	–	1 (0.3)	–	9 (2.4)	–	21 (5.7)
Days 31–92	11 (3.0)	3 (0.8)	–	–	15 (4.1)	–	29 (7.9)
Days 93–183	11 (3.0)	7 (1.9)	2 (0.5)	1 (0.3)	25 (6.8)	1 (0.3)	47 (12.8)
Days 184–274	7 (1.9)	6 (1.6)	–	1 (0.3)	13 (3.5)	1 (0.3)	28 (7.6)
Days 275–364	7 (1.9)	5 (1.4)	–	–	8 (2.2)	–	20 (5.4)
Total	47 (12.8)	21 (5.7)	3 (0.8)	2 (0.5)	70 (19.0)	2 (0.5)	145 (39.4)
Revefenacin 175 μg	(*n* = 335)						
Days 1–30	6 (1.8)	1 (0.3)	–	–	11 (3.3)	–	18 (5.4)
Days 31–92	11 (3.3)	2 (0.6)	–	–	28 (8.4)	1 (0.3)	42 (12.5)
Days 93–183	11 (3.3)	5 (1.5)	2 (0.6)	–	24 (7.2)	–	42 (12.5)
Days 184–274	6 (1.8)	5 (1.5)	–	–	10 (3.0)	–	21 (6.3)
Days 275–364	8 (2.4)	4 (1.2)	1 (0.3)	–	7 (2.1)	1 (0.3)	21 (6.3)
Total	42 (12.5)	17 (5.1)	3 (0.9)	–	80 (23.9)	2 (0.6)	144 (43.0)
Tiotropium 18 μg	(*n* = 357)						
Days 1–30	3 (0.8)	–	–	–	3 (0.8)	–	6 (1.7)
Days 31–92	6 (1.7)	2 (0.6)	–	1 (0.3)	10 (2.8)	–	19 (5.3)
Days 93–183	8 (2.2)	3 (0.8)	–	–	14 (3.9)	–	25 (7.0)
Days 184–274	7 (2.0)	5 (1.4)	–	–	9 (2.5)	–	21 (5.9)
Days 275–364	9 (2.5)	5 (1.4)	1 (0.3)	–	7 (2.0)	1 (0.3)	23 (6.4)
Total	33 (9.2)	15 (4.2)	1 (0.3)	1 (0.3)	43 (12.0)	1 (0.3)	94 (26.3)

*AE* adverse event

The percentage of subjects who discontinued treatment due to adverse events (AEs) was similar in both revefenacin groups (12–13%) and lower in the tiotropium group (9%). However, with respect to the most commonly reported AEs, a similar percentage of subjects across groups (< 2.5%) discontinued study drug due to COPD exacerbation, whereas more subjects in the revefenacin groups (1.8% and 2.5% in the 175 μg and 88 μg groups, respectively) discontinued due to dyspnea compared with the tiotropium group (0.6%).

### Efficacy

**Fig. 1:**
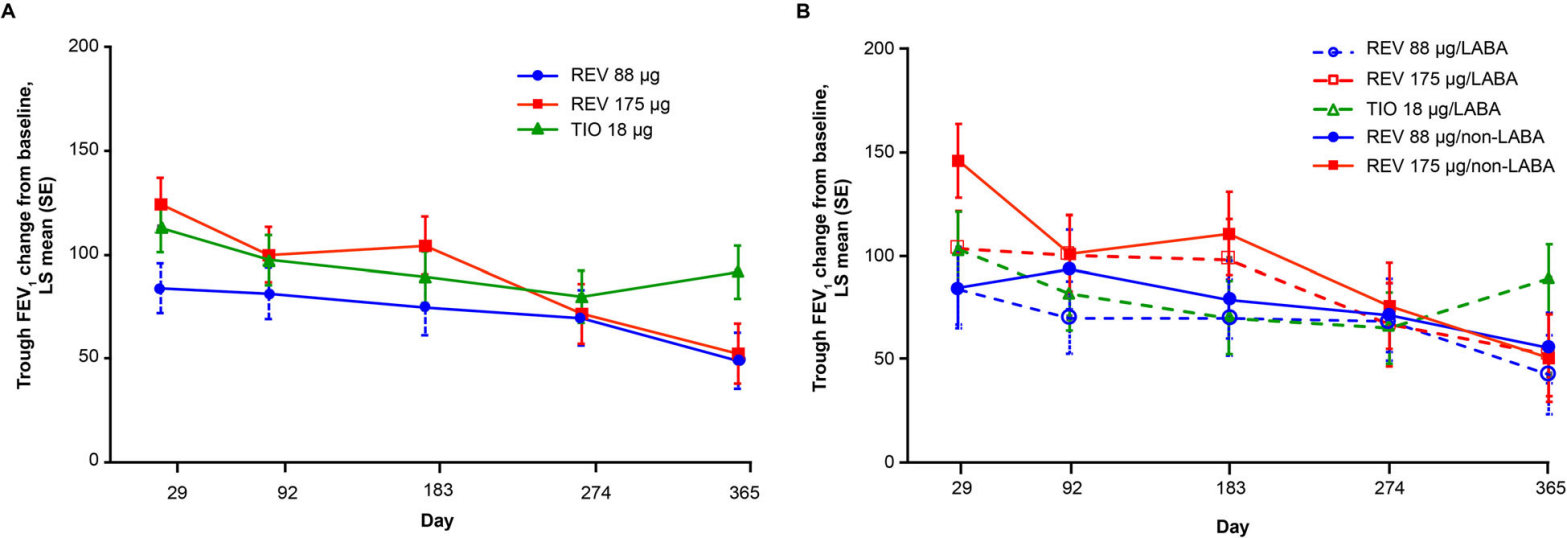
Trough FEV_1_ (mL) change from baseline during the 1-year treatment period (**a**) for the overall study population and (**b**) in subgroups of patients with or without concurrent use of LABA (LS mean change from baseline; ITT population). FEV_1_, forced expiratory volume in 1 s; ITT, intent-to-treat; LABA, long-acting β-agonist; LS, least squares; REV, revefenacin; SE, standard error; TIO, tiotropium

Over the 52-week treatment period, both doses of revefenacin, as well as tiotropium, elicited statistically significant (all *p* < 0.0003) improvements from baseline in trough FEV_1_ (Table 3, Fig. 1a). The trough FEV_1_ profile for revefenacin 175 μg (range, 52.3–124.3 mL) was similar to that of tiotropium (range, 79.7–112.8 mL) up to Month 9, but diverged after that, in part due to differences in subject discontinuation in the final 3 months of the trial (Table 4).

**Table 3:** Change from baseline in trough FEV_1_ (mL) during the 1-year treatment period

Trough FEV_1_ (mL), LS mean (95% CI)	Revefenacin 88 μg	Revefenacin 175 μg	Tiotropium 18 μg
Day 29	83.8 (60.4, 107.1) *n* = 317	124.3 (99.5, 149.1) *n* = 282	112.8 (89.8, 135.8) *n* = 330
Day 92	81.3 (57.1, 105.5) *n* = 287	100.0 (73.9, 126.1) *n* = 243	97.3 (73.7, 120.9) *n* = 307
Day 183	74.2 (48.6, 99.8) *n* = 239	104.4 (77.1, 131.7) *n* = 210	89.0 (64.9, 113.2) *n* = 283
Day 274	69.5 (43.4, 95.6) *n* = 223	71.4 (43.2, 99.6) *n* = 189	79.7 (55.0, 104.3) *n* = 265
Day 365	48.8 (22.3, 75.3) *n* = 212	52.3 (23.9, 80.6) *n* = 185	91.5 (66.4, 116.5) *n* = 248

Data are mean (standard deviation)

*CI* confidence interval, *FEV*_*1*_ forced expiratory volume in 1 s, *LS* least squares

**Table 4:** Change from baseline in trough FEV_1_ (mL), according to patient withdrawal, during the 1-year treatment period

Trough FEV_1_ (mL), LS mean (SE)	Revefenacin 88 μg	Revefenacin 175 μg	Tiotropium 18 μg
Day 29 and still on study at Day 92	88.8 (9.63) *n* = 278	121.8 (11.10) *n* = 232	113.7 (8.64) *n* = 296
Day 92 and still on study at Day 183	84.5 (10.74) *n* = 236	100.8 (11.78) *n* = 203	104.7 (8.88) *n* = 275
Day 183 and still on study at Day 274	73.1 (11.50) *n* = 216	98.9 (12.27) *n* = 182	94.4 (9.13) *n* = 259
Day 274 and still on study at Day 365	68.3 (11.46) *n* = 207	65.6 (11.71) *n* = 181	86.1 (9.21) *n* = 242
Day 29 and withdrew prior to Day 92	73.4 (19.25) *n* = 39	130.8 (15.48) *n* = 50	130.8 (21.37) *n* = 34
Day 92 and withdrew prior to Day 183	93.1 (19.21) *n* = 51	97.3 (26.50) *n* = 40	42.7 (25.92) *n* = 32
Day 183 and withdrew prior to Day 274	99.4 (33.22) *n* = 23	137.2 (34.49) *n* = 28	143.5 (32.19) *n* = 24
Day 274 and withdrew prior to Day 365	83.4 (55.68) *n* = 16	60.0 (106.42) *n* = 8	57.0 (36.85) *n* = 23

*FEV*_*1*_ forced expiratory volume in 1 s, *LS* least squares, *SE* standard error

**Fig. 2:**
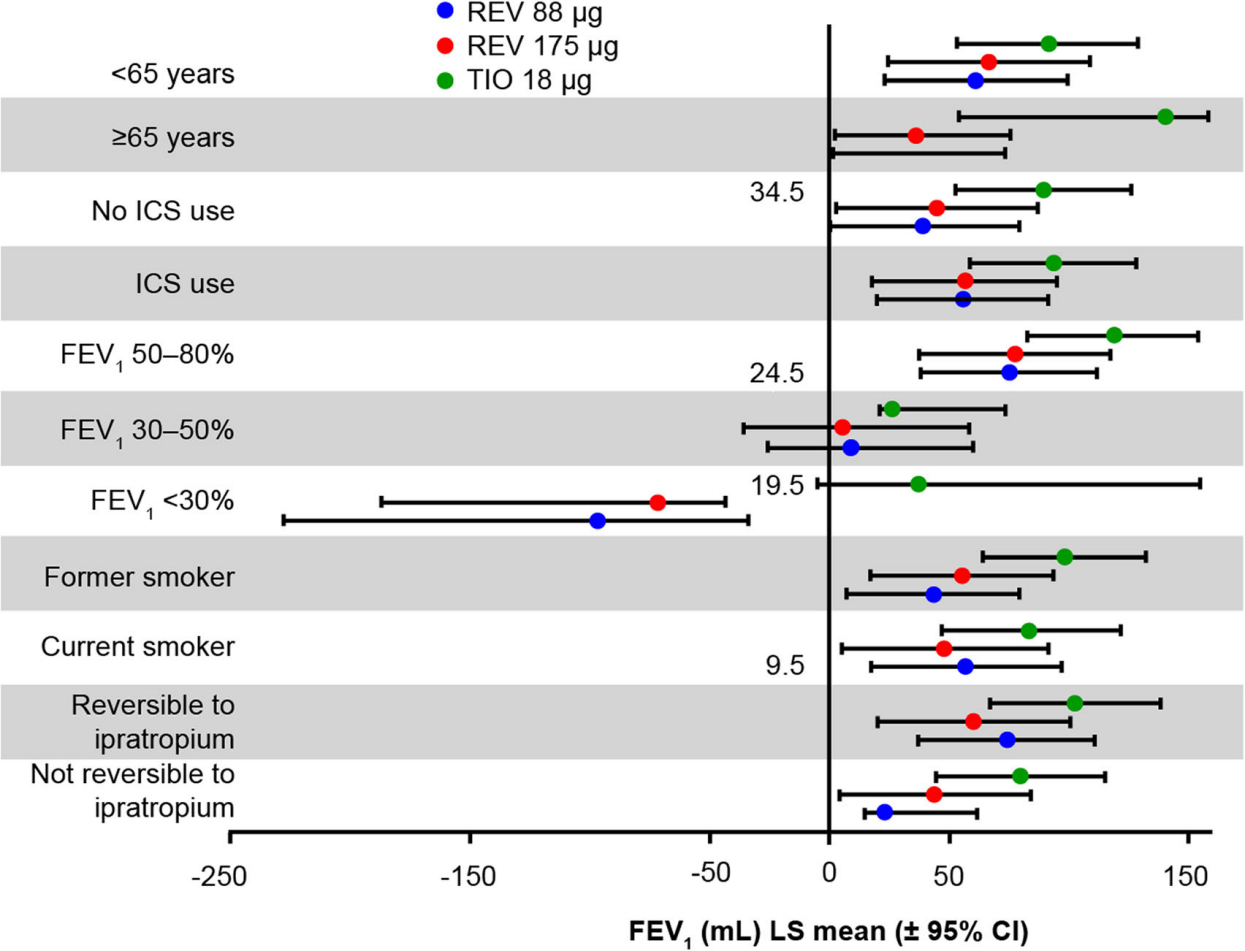
Day 365 trough FEV_1_ by patient subgroup. CI, confidence interval; FEV_1_, forced expiratory volume in 1 s; ICS, inhaled corticosteroid; LS, least squares; REV, revefenacin; TIO, tiotropium

In subgroup comparisons, the effects on trough FEV_1_ among patients who were using a LABA ranged from 51.8–102.9 mL with revefenacin 175 μg, and 64.7–102.5 mL with tiotropium (Fig. 1b). All subgroups responded similarly overall in the three treatment arms (Fig. 2).

### Health outcomes assessments

**Fig. 3:**
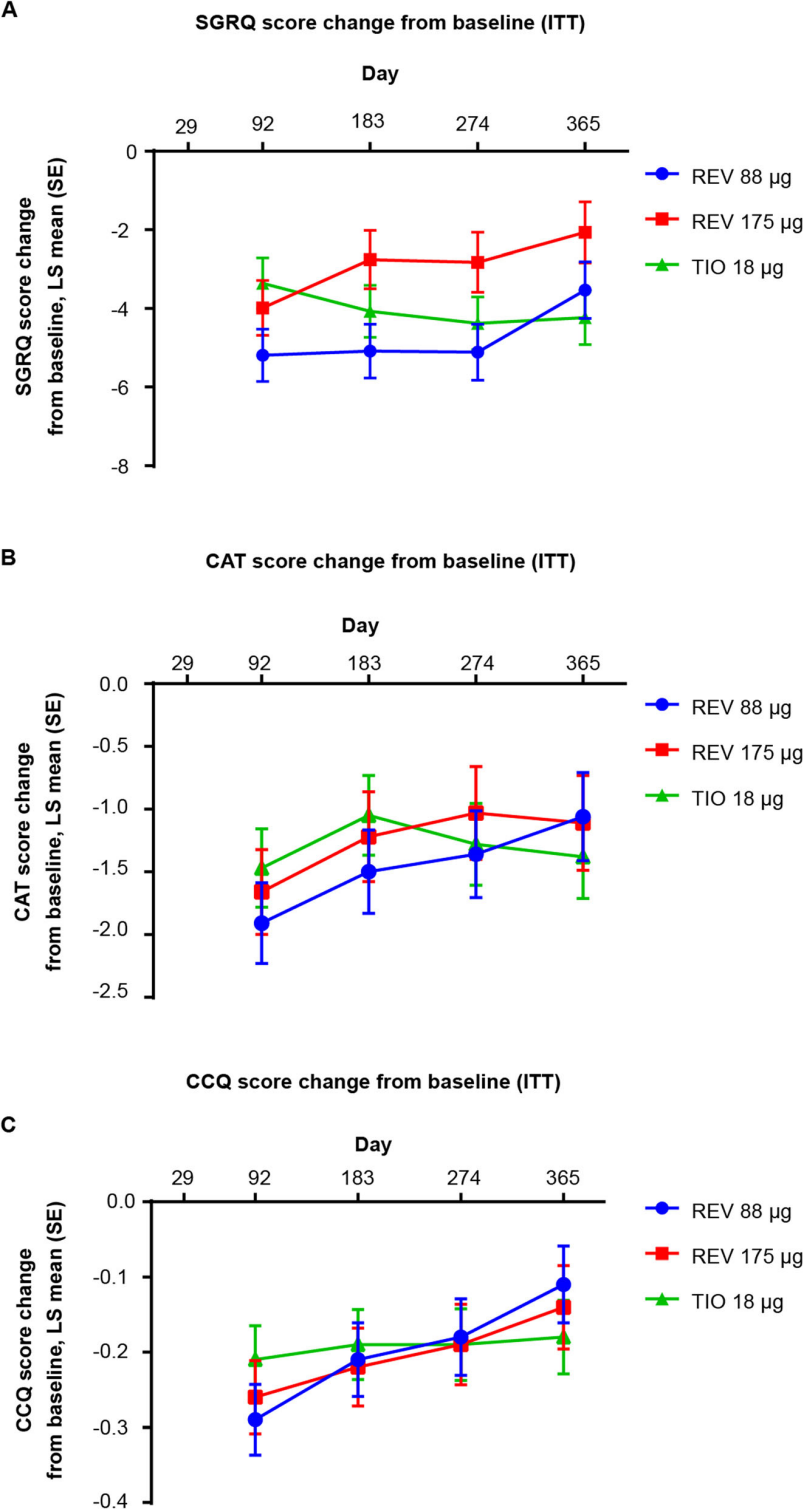
LS mean change in (**a**) SGRQ, (**b**) CAT and (**c**) CCQ from baseline for health outcomes assessments. For each health outcomes assessment, all treatment groups showed statistically significant (*p* < 0.05) changes from baseline at all time points. CAT, COPD Assessment Test; CCQ, Clinical COPD Questionnaire; COPD, chronic obstructive pulmonary disease; ITT, intent-to-treat; LS, least squares; REV, revefenacin; SE, standard error; SGRQ, St. George’s Respiratory Questionnaire; TIO, tiotropium

There was a statistically significant (*p* < 0.05) improvement in SGRQ, CAT and CCQ at all time points assessed from 3 months for all three treatment arms (Fig. 3). Analysis of MCID response based on SGRQ total score at Day 365 revealed that there was a similar percentage of responders in the tiotropium, revefenacin 175 μg and revefenacin 88 μg groups (53%, 42% and 45% respectively). The percentage of CAT responders in all three treatment groups were similar (revefenacin 175 μg [48%] revefenacin 88 μg group [43%] and tiotropium group [47%]). Changes in CAT and CCQ scores did not reach the pre-determined thresholds for clinical significance in any group at any time point.

The revefenacin 175 μg group had 153 COPD exacerbations and the rate of exacerbations (1.78; 95% CI: 1.37, 2.30) during the year of treatment was based on data from the EXACT-PRO tool. The lower-dose revefenacin and tiotropium groups reported 197 and 192 exacerbations, and exacerbation rates of 2.46 (95% CI: 1.93, 3.15) and 2.31 (95% CI: 1.78, 2.99), respectively. Mean exacerbation severity scores were similar between treatment groups (53 to 54 on a 0 to 100 scale, in which higher scores indicate more severe disease). Data from the EXACT-PRO tool supported the study’s findings based on evaluation of COPD exacerbations reported as treatment-emergent AEs [8].

Evaluation of breathlessness using the TDI total score, in which lower scores represent worsening dyspnea, indicated a decrease in breathlessness in all treatment groups throughout the study. TDI scores in the revefenacin groups and tiotropium group were (LS mean TDI total score at Day 365) 0.92, 1.37 and 0.87 in the revefenacin 175 μg, revefenacin 88 μg and tiotropium groups, respectively. The percentage of TDI responders between treatment groups at Day 365 were 85/184 patients [LS proportion 43.8%], 110/211 patients [50.9%] and 103/244 [40.3%] in the revefenacin 175 μg, revefenacin 88 μg and tiotropium groups, respectively.

Evaluation of rescue albuterol use showed an average of LS mean (standard error) values of 1.6 (0.23), 1.9 (0.2) and 1.3 (0.21) puffs per day over the 12-month treatment period in the revefenacin 175 μg, revefenacin 88 μg, and tiotropium groups, respectively. However, there was a consistent trend toward a decrease in puffs per day in all treatment groups throughout the study. The percentage of rescue-free days was similar across all groups (average LS mean percentage of rescue-free 24-h periods over the 12-month treatment period: 50–54%).

Overall treatment satisfaction was similar at the beginning and end of treatment and across treatment groups at both time points measured.

## Discussion

Maintained therapeutic effect during 1 year of once-daily treatment with revefenacin 175 μg and 88 μg, and tiotropium 18 μg is described. While the study was not powered to show differences between treatments, and is complicated by the bias introduced through open-label use of an active comparator, the report demonstrates statistically significant improvements in lung function measured by change from baseline in trough FEV_1_ for both revefenacin dose groups and tiotropium over the entire 52-week treatment period. In addition, health outcomes assessments used to measure the severity of disease-specific symptoms (SGRQ, CAT, CCQ and BDI/TDI), and analysis of rescue medication use, demonstrated statistically significant improvements from baseline. Mean exacerbation severity scores were similar between treatment groups. The safety of revefenacin has been previously demonstrated [8].

The decline in lung function in all three treatment groups over the course of the 52-week trial reflects disease progression and is expected in any long-term efficacy analysis of COPD treatments. Interestingly, lung function in the tiotropium group appeared to improve during the final 3 months of treatment. The increase in FEV_1_ observed on Day 365 in the tiotropium group may have been the result of the disproportionate number of poor performers (assessed by trough FEV_1_) who discontinued tiotropium during the final 3 months of treatment. The effect of revefenacin on trough FEV_1_ in this trial is consistent with that seen in previous studies [7, 13].

Analysis of withdrawal rates indicated that a higher proportion of subjects withdrew from both revefenacin arms than the tiotropium arm overall and during each 3-month treatment period. The slightly higher overall rate of AE-related withdrawals, in particular withdrawals due to dyspnea (1.8% and 2.5% in the revefenacin 175 μg and 88 μg groups, respectively, versus 0.6% in the tiotropium group) may explain this. The open-label design for the tiotropium treatment arm may have also influenced withdrawal rates. There is no evidence from the FEV_1_ data that patients withdrew due to lack of treatment efficacy. As the study population is comprised of a considerable amount of COPD patients with severe markers of disease (FEV_1_ < 50%, mMRC ≥2 or CAT ≥10), it is possible these patients with more severe disease withdrew from the study at different rates. This would lead to a bias in the results, which is mainly driven by the open label nature of the study design. The dropout rate seen with revefenacin was similar to that seen in 12-month trials with other nebulized long-acting bronchodilators [14, 15].

With regard to patient subgroups, the concurrent LABA users in all treatment groups demonstrated significant improvements from baseline in trough FEV_1_. This result is comparable with the phase 3 revefenacin studies [7]. These findings are important to note as dual bronchodilation is increasingly recommended by COPD treatment guidelines [1]. Other subgroups (baseline smoking status, age category, current ICS use, responsiveness to ipratropium, and GOLD airflow) were also assessed to evaluate the consistency of treatment effects on trough FEV_1_ across a wider range of the ITT population. All three treatment groups achieved nominal improvements in trough FEV_1_ compared with baseline values. Overall, these subgroup results were similar with the phase 3 revefenacin studies [7].

Study limitations include lack of the open-label design for the tiotropium treatment arm, which likely led to different withdrawal rates for the revefenacin and tiotropium arms, and therefore skewed the efficacy results. In addition, the ability to draw conclusions on the efficacy of revefenacin versus tiotropium was limited because the study was not designed or powered to demonstrate statistically significant differences between treatment arms. Larger studies that are powered to assess efficacy are required to assess the comparative effects of these two treatments. However, the comparability of the results over 52 weeks is assuring.

## Conclusion

In this study, which included a trial population with a broad range of disease severity, significant sustained improvements from baseline in trough FEV_1_ and respiratory health outcomes were demonstrated for the revefenacin 175 μg dose and open-label tiotropium over 52 weeks. In line with the FDA approval for revefenacin 175 μg, these data further supports the use of revefenacin 175 μg as a once-daily bronchodilator for the nebulized treatment of patients with COPD.

## Supplementary information

**Additional file 1: Table S1.** Patients who discontinued by study day: change from baseline in trough FEV_1_ (mL) at last assessment before withdrawal.

## Data Availability

Theravance Biopharma (and its affiliates) will not be sharing individual de-identified participant data or other relevant study documents.

## Abbreviations

AE: Adverse event
BDI/TDI: Baseline and Transition Dyspnea Index
BMI: Body mass index
CAT: COPD Assessment Test
CCQ: Clinical COPD Questionnaire
CI: Confidence interval
COPD: Chronic obstructive pulmonary disease
EXACT-PRO: EXAcerbation of Chronic Pulmonary Disease Tool-Patient Related Outcome
FEV_1_: Forced expiratory volume in 1 s
FVC: Forced vital capacity
GOLD: Global Initiative for Chronic Obstructive Lung Disease
ICS: Inhaled corticosteroid
ITT: Intent-to-treat
LABA: Long-acting β-agonist
LAMA: Long-acting muscarinic antagonist
LS: Least squares
MCID: Minimal clinically important difference
mMRC: modified Medical Research Council
REV: Revefenacin
RMMM: Repeated measures mixed-effect model
SD: Standard deviation
SE: Standard error
SGRQ: St. George’s Respiratory Questionnaire
TIO: Tiotropium
y: years

## References

[1] GOLD: Global Initiative for Chronic Obstructive Lung Disease. 2018.

[2] HIGHLIGHTS OF PRESCRIBING INFORMATION LONHALA™ MAGNAIR™ (glycopyrrolate) inhalation solution, for oral inhalation [https://www.accessdata.fda.gov/drugsatfda_docs/label/2017/208437lbl.pdf].

[3] HIGHLIGHTS OF PRESCRIBING INFORMATION YUPELRI® (revefenacin) inhalation solution, for oral inhalation [https://www.accessdata.fda.gov/drugsatfda_docs/label/2018/210598s000lbl.pdf].

[4] Steinfeld T, Pulido-Rios MT, Chin K, King K, Huang JX, Lee TW, Jasper JR, Ji Y, Hegde S, Mammen M (2009). In vitro characterization of TD-4208, a lung-selective and long-acting muscarinic antagonist bronchodilator [abstract]. Am J Respir Crit Care Med.

[5] Baldwin M, McConn D, Potgieter P, Steinfeld T, Quinn D, Moran E (2013). Single-dose pharmacokinetics of TD-4208, a novel long-acting muscarinic antagonist, in patients with COPD [abstract]. Am J Respir Crit Care Med.

[6] Quinn D, Barnes CN, Yates W, Bourdet DL, Moran EJ, Potgieter P, Nicholls A, Haumann B, Singh D (2018). Pharmacodynamics, pharmacokinetics and safety of revefenacin (TD-4208), a long-acting muscarinic antagonist, in patients with chronic obstructive pulmonary disease (COPD): results of two randomized, double-blind, phase 2 studies. Pulm Pharmacol Ther.

[7] Ferguson G, Feldman G, Pudi K, Barnes C, Moran E, Haumann B, Pendyala S, Crater G (2019). Improvements in lung function with nebulized Revefenacin in the treatment of patients with moderate to very severe COPD: results from two replicate phase III clinical trials. Chronic Obstr Pulm Dis.

[8] Donohue J, Kerwin E, Sethi S, Haumann B, Pendyala S, Dean L, Barnes C, Moran E, Crater G (2019). Revefenacin, a once-daily, lung-selective, long-acting muscarinic antagonist for nebulized therapy: safety and tolerability results of a 52-week phase 3 trial in moderate to very severe chronic obstructive pulmonary disease. Respir Med.

[9] Integrated addendum to ICH harmonised guideline: guideline for good clinical practice E6 (R2) [https://goo.gl/CFOmR3].

[10] Helsinki (2013). World medical association declaration of Helsinki: ethical principles for medical research involving human subjects. JAMA.

[11] Spirometry for health care providers [https://goldcopd.org/wp-content/uploads/2016/04/GOLD_Spirometry_2010.pdf].

[12] Leidy N, Murray L, Jones P, Sethi S. Performance of the EXAcerbations of chronic pulmonary disease tool patient-reported outcome measure in three clinical trials of chronic obstructive pulmonary disease. Ann Am Thorac Soc. 2014;(3):316–25. 10.1513/AnnalsATS.201309-305OC 24432712

[13] Pudi KK, Barnes CN, Moran EJ, Haumann B, Kerwin E (2017). A 28-day, randomized, double-blind, placebo-controlled, parallel group study of nebulized revefenacin in patients with chronic obstructive pulmonary disease. Respir Res.

[14] Donohue J, Hanania N, Fogarty C, Campbell S, Rinehart M, Denis-Mize K. Long-term safety of nebulized formoterol: results of a twelve-month open-label clinical trial. Ther Adv Respir Dis. 2008;(4):199–208. 10.1177/1753465808093934 19124372

[15] Donohue J, Hanania N, Sciarappa K, Goodwin E, Grogan D, Baumgartner R, Hanrahan J (2008). Arformoterol and salmeterol in the treatment of chronic obstructive pulmonary disease: a one year evaluation of safety and tolerance. Ther Adv Respir Dis.

