# Supplementary material for: Maintained therapeutic effect of revefenacin over 52 weeks in moderate to very severe Chronic Obstructive Pulmonary Disease (COPD)
Source: Respir Res. 2019 Oct 30;20:241. doi: 10.1186/s12931-019-1187-7 (PMC6822411; doi:10.1186/s12931-019-1187-7)
Supplement: Supplementary file 1 — Additional file 1: Table S1. Patients who discontinued by study day: change from baseline in trough FEV1 (mL) at last assessment before withdrawal. [file 12931_2019_1187_MOESM1_ESM.docx]

**SUPPLEMENTARY INFORMATION**

**Additional file 1: Table S1.** Patients who discontinued by study day: change from baseline in trough FEV_1_ (mL) at last assessment before withdrawal

|  | **Revefenacin 88 µg**  **(*n* = 141)** | **Revefenacin 175 µg**  **(*n* = 138)** | **Tiotropium 18 µg**  **(*n* = 88)** |
| --- | --- | --- | --- |
| Day 93 | 70.0 (169.5) | 123.5 (185.6) | 84.2 (143.9) |
| Day 184 | 100.1 (201.1) | 106.4 (245.0) | 102.1 (234.5) |
| Day 275 | 14.0 (233.1) | 217.7 (283.9) | 43.0 (234.8) |
| Day 365 | 90.4 (255.0) | 130.1 (264.0) | 59.9 (285.3) |

Data are raw mean (standard deviation). FEV_1_, forced expiratory volume in 1 second.
